# The Application and Optimisation of a Neural Network PID Controller for Trajectory Tracking Using UAVs

**DOI:** 10.3390/s24248072

**Published:** 2024-12-18

**Authors:** Michał Siwek, Leszek Baranowski, Edyta Ładyżyńska-Kozdraś

**Affiliations:** 1 Faculty of Mechatronics, Armament and Aerospace, Military University of Technology, Kaliskiego 2 Street, 00-908 Warsaw, Poland; leszek.baranowski@wat.edu.pl; 2 Faculty of Mechatronics, Warsaw University of Technology, ul. św. Boboli 8, 02-525 Warsaw, Poland; edyta.ladyzynska@pw.edu.pl

**Keywords:** UAV, trajectory tracking, path tracking, pitch channel control, PID tuning, neural network, Ziegler–Nichols II method

## Abstract

This paper considers the problem of flying a UAV along a given trajectory at speeds close to the speed of sound and above. A novel pitch channel control system is presented using the example of a trajectory with rapid and large changes in flight height. The control system uses a proportional–integral–differential (PID) controller, whose gains were first determined using the Ziegler-Nichols II method. The determined gains were then optimised to minimise height error using a recurrent back-propagation neural network (PIDNN), with which new controller gains were determined, which is also a novelty of this study. Simulations were carried out for flights at subsonic speeds close to the speed of sound and supersonic speeds, at low and high altitudes. The simulations showed that determining controller gains using a recurrent neural network significantly minimises height errors and increases the flexibility of the PID controller.

## 1. Introduction

Unmanned aerial vehicles (UAVs) represent one of the fastest growing technologies in the modern world and are of great interest to researchers. Since their first military applications, UAVs have been widely used in many fields, including agriculture, surveying, logistics, rescue, scientific research, and entertainment. Advances in component miniaturisation, communication technology development, navigation systems and artificial intelligence have made drones increasingly advanced, versatile, and accessible. UAVs have many unique advantages, such as the ability to reach disaster areas, fast mobility, aerial missions, and transport cargo. Despite these numerous advantages, they face some limitations related to flight autonomy, path planning, battery life, battery endurance and a limited ability to carry heavy payloads, making it difficult to charge them with higher-powered supplies [1].

Developments in control technology have led to many controller solutions for flight stabilisation and trajectory tracking. Controllers mainly include model predictive control [2,3], sliding mode control [4,5,6] LQR control [7], backstepping control [8,9] and (the most widely used) PID control [10,11,12,13,14,15,16,17].

The control methods encountered in the literature mainly concern UAVs that implement trajectories at heights of several metres at speeds significantly lower than the speed of sound. A unique and difficult case is the control of UAVs that fly at speeds close to the speed of sound, a topic that is not often encountered in the literature. When flying at speeds close to the speed of sound, the physical properties of the UAV airframe change non-linearly over time and depend, among other things, on the speed and height of flight [18]. Changes in these properties can also be caused by external disturbances. This means that the system in question is non-stationary and random. In turn, the non-stationarity of the static and dynamic characteristics of the airframe determines changes in the characteristics of the entire control loop, which in extreme cases can threaten its stability. This is a highly undesirable effect that has a negative impact on trajectory tracking accuracy. To compensate for the influence of the airframe’s dynamic characteristics and adverse flight conditions, an autopilot is installed on board the UAV as a stabilising device.

Supersonic UAVs are an area of continuous research and experimentation by various organisations, mainly in military applications. Defence and aerospace manufacturers are involved in research projects to develop supersonic UAVs. For example, companies such as Boeing and Lockheed Martin have been exploring concepts for unmanned systems that can reach supersonic speeds. Some of their prototypes have been tested, focusing on aerodynamics, control systems, propulsion systems and materials that can withstand supersonic flight conditions. These tests help inform design decisions and verify their claimed performance [19].

The primary advantage is their ability to travel faster than the speed of sound, significantly reducing travel time for reconnaissance, surveillance, or delivery missions. In military and emergency scenarios, supersonic UAVs can reach target areas quickly, providing real-time intelligence and support. Their high speed allows for quick coverage of large areas, which is beneficial for monitoring and reconnaissance tasks. Supersonic UAVs can quickly penetrate hostile airspace, potentially avoiding detection for longer periods, especially if equipped with stealth features. The speed and altitude of supersonic UAVs might allow for carrying more advanced sensors and equipment, improving the quality of data collected. While these advantages present exciting possibilities, their practical implementation must overcome significant technical, regulatory, and economic challenges [18,19,20].

The novelty of this paper is the development of a method to control the yaw channel of an unmanned aircraft that is flying close to the speed of sound and above. This problem is described in more detail in the Related Work section and has not yet been discussed in the literature. This paper considers an example of flight to a designated point along a desired trajectory, given a sudden and significant change in flight height while maintaining speed. A classical approach using the Ziegler–Nichols II method was applied for the first time in the synthesis of a control system. The control coefficients selected in this way had to be optimised in order to minimise errors at the final trajectory stage. For optimisation, the author’s approach of using a recursive neural network to tune the controller was used, which is also a novelty of this article. In summary, this paper presents a novel autopilot design for an unmanned aerial vehicle using a PID controller (PIDNN) with a neural network.

The remainder of this article is organised as follows: Section 2 discusses the research problem and presents a mathematical model of the research object. Section 3 presents a literature analysis of UAV autopilots. Section 4 presents the control system design and methods for determining the gains of the PID and PIDNN controllers. Section 5 covers flight simulations along a desired trajectory and a discussion of the results. Finally, this study is summarised in Section 6.

## 2. Modelling and Problem Formulation

The movement of controlled objects is usually simplified to the popular task of tracking the path. This task is solved in the case of land-based mobile robots, drones, and other unmanned aerial vehicles that can perform flights at very high altitudes (on the order of several kilometres) and even greater distances (on the order of tens and often hundreds of kilometres). Of particular interest are objects that reach very high flight speeds, often approaching or exceeding the speed of sound. It is these UAVs that are the focus of this article. A mathematical model of the UAV considered in this article is presented below, as well as an example flight scenario from which the research problem addressed in this article was formulated.

### 2.1. Modelling of the Object

**Figure 1:**
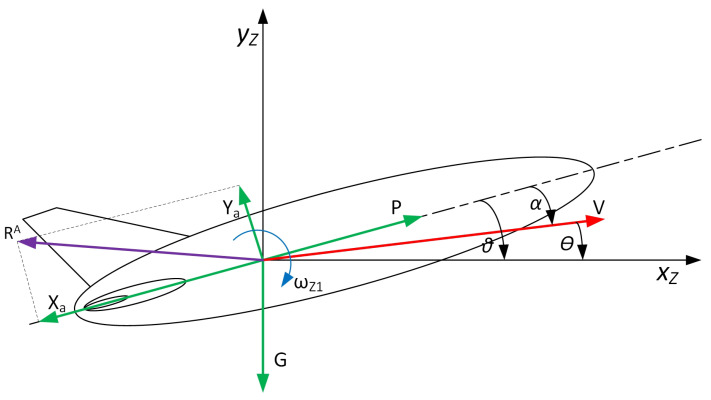
Cross-section in the control plane of the UAV under consideration.

A scheme of the UAV considered in this article is shown in Figure 1.

where: *V*—linear velocity, $\omega_{Z_{1}}$—angular rate, *P*—the force of the thrust, $X_{a}$—drag force, $Y_{a}$—lifting force, *G*—Gravity, Θ—angle of the inclination of the velocity vector, α—angle of attack, ϑ—UAV pitch angle, $x_{Z}$, $y_{Z}$-coordinates of the UAV in the Earth’s coordinate system.

To analyse the parameters of the trajectory tracking control system being developed, simplifying assumptions were made to construct a mathematical model of the research object as follows:

- The UAV is considered as a rigid body with six degrees of freedom;
- The flight occurs in a standard atmosphere as described by the ISA model;
- The UAV is stabilised at the pitch angle; the vertical plane is controlled by the elevator $\delta_{w}$;
- The maximum angle of the elevator is $\delta_{max}$ = 15°.

The mathematical model of the UAV was assumed to be a system of non-linear equations of longitudinal motion in the Earth’s coordinate system:

(1) $$\frac{dV}{dt}=\frac{1}{m}\left(Pcos\alpha -X_{a}-Gsin\Theta \right)$$

(2) $$\frac{d\Theta }{dt}=\frac{1}{mV}\left(Psin\alpha -Y_{a}-Gcos\Theta \right)$$

(3) $$\frac{d\omega_{z_{1}}}{dt}=\frac{M_{Z_{1}}}{I_{Z_{1}}}$$

(4) $$\frac{d\vartheta }{dt}=\omega_{z_{1}}$$

(5) ϑ=α+Θ

(6) $$\frac{dx_{z}}{dt}=Vcos\Theta$$

(7) $$\frac{dy_{z}}{dt}=Vsin\Theta$$

where: *m*—mass, $I_{Z_{1}}$—moment of inertia, $M_{Z_{1}}$—tilting torque, and additional equations:

(8) $$X_{a}=C_{X_{\alpha }}\frac{\rho V^{2}}{2}S$$

(9) $$C_{X_{a}}=C_{X_{\alpha }}^{\alpha^{2}}\alpha^{2}+C_{X_{\alpha }}^{{\delta_{w}}^{2}}{\delta_{w}}^{2}+C_{X_{0}}$$

(10) $$Y_{a}=C_{Y_{\alpha }}\frac{\rho V^{2}}{2}S$$

(11) $$C_{Y_{a}}=C_{Y_{\alpha }}^{\alpha }\alpha +C_{Y_{\alpha }}^{\delta_{w}}\delta_{w}$$

(12) $$M_{Z_{1}}=m_{Z_{1}}\frac{\rho V^{2}}{2}SL$$

(13) $$m_{Z_{1}}=m_{Z_{1}}^{\alpha }\alpha +m_{Z_{1}}^{\delta_{w}}\delta_{w}+m_{Z_{1}}^{{\bar{\omega }}_{Z_{1}}}{\bar{\omega }}_{Z_{1}}$$

(14) $${\bar{\omega }}_{Z_{1}}=\frac{\omega_{Z_{1}}L}{V}$$

where: $C_{X_{a}}$—drag coefficient, $C_{X_{0}}$, $C_{X_{a}}^{\alpha^{2}}$, $C_{X_{a}}^{{\delta_{w}}^{2}}$—aerodynamic factors related to drag force, $C_{Y_{a}}$—lift coefficients, $C_{Y_{a}}^{\alpha }$, $C_{Y_{a}}^{\delta_{w}}$—aerodynamic factors related to lift force, $\delta_{w}$—steering angle, ρ—atmospheric density, *S*—characteristic surface, *L*—characteristic linear parameter, $m_{Z_{1}}$, $m_{Z_{1}}^{\alpha }$, $m_{Z_{1}}^{\delta_{w}}$, $m_{Z_{1}}^{\omega_{Z_{1}}}$—coefficients related to the Mach number.

### 2.2. Problem Statement

**Figure 2:**
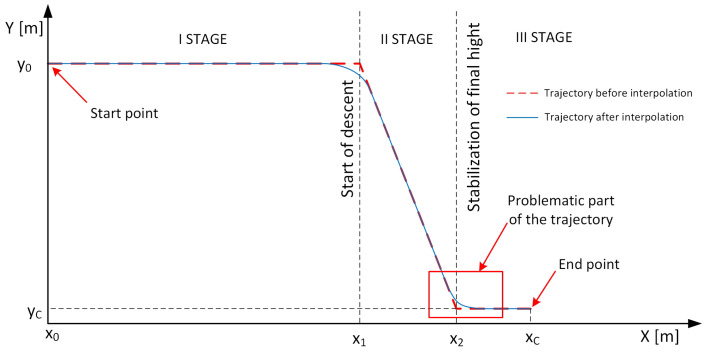
Example trajectory of the UAV under consideration.

An example of the trajectory of the UAV under consideration is shown in Figure 2. The choice of this trajectory was based on the observed lack of research in this area and the problems described in the articles [21,22,23] related to the UAV’s tilt-down and tilt-up manoeuvres. This comes down to control of the pitching moment, namely the pitch control channel, which is a major challenge given flight speeds close to the speed of sound.

The UAV movement was assumed to be on a vertical plane aligned with the control plane. On the *X*-axis, the distance from the starting point to significant points of the trajectory is marked. On the *Y*-axis, the UAV’s flight height is marked. The end point to be reached by the object is $x_{c}$ = 120 km from the starting point of the flight $x_{0}$. Therefore, the article will consider the pitch control channel of the UAV. The first stage of the trajectory assumes flight at a preset height and maintaining a preset speed. The second stage is a flight descent manoeuvre to a height of 4 m, which ends within $x_{2}$ = 5 km from the final point of the trajectory. The third stage involves flying to a height of $y_{C}$ = 4 m above the surface of water or land. The descent manoeuvre should be executed as quickly as possible and end as close to the end point $x_{c}$ as possible. This raises the need to develop a controller characterised by very short regulation times and very low transient errors. This aspect is particularly important when stabilising the flight of a UAV at very low heights (on the order of a few metres), as high values of transient errors can cause the UAV to hit the surface of water or the ground, far from the end point (which is highlighted in the Figure 2 as a problematic part of the trajectory).

Simplifying the described issue to the task of tracking a path by a UAV, it is expected that the angular velocity $\omega_{Z_{1}}$ will be approximately equal to the angular velocity $\dot{\Theta }$ of the UAV velocity vector, which means the following:

(15) $$\omega_{Z_{1}}=\dot{\Theta }$$

Therefore, the task of the controller in the UAV pitch channel is to minimise the quantitative index in the form of

(16) $$J=\int_{0}^{t}\left|\omega_{Z_{1}}-\dot{\Theta }\right|dt$$

## 3. Related Works

Controlling movement along a desired path is one of the fundamental problems in the development of autonomous unmanned systems. The dynamics of unmanned systems is highly related to non-linearity, with uncertainties due to modelling errors [24,25,26,27] and time-varying external disturbances [9]. A review of the literature reveals different methods for controlling spatial positions. Some works use feedback controllers separately for each channel, neglecting the coupling between angles, while others solve the problem directly using non-linear control approaches. For example, Li et al. [28] proposed a longitudinal non-linear control law design for UAVs consisting of a combination of a Takagi and Sugeno (T-S) fuzzy model and guaranteed cost control (GCC) technique. The basic concept of the T-S fuzzy model is to approximate a complex non-linear system as a collection of several local linear subsystems, integrating these subsystems using fuzzy logic to effectively control the original system. GCC offers a methodology for designing feedback controllers in uncertain systems. By taking into account uncertainties in the system and aiming to minimise the cost function, GCC ensures reliable system operation. The GCC controller features a simple design and convenient parameter tuning. Its robustness and ability to reject interference make it well suited for controlling MIMO systems. Within the fuzzy T-S model, a strict stability criterion was given using linear matrix inequalities. Then, using the GCC technique, an extended state feedback controller was designed to achieve good robustness and interference rejection capability. Finally, the longitudinal controller designed using the F-GCC technique shows better transient response and better robustness in the presence of disturbances.

Another common approach to UAV control is sliding-mode control methods. Kuang and Chen in [6] presented a sliding-mode adaptive control approach with an inner and outer loop structure, increasing robustness and adaptability. In this approach, adaptive laws are introduced to manage uncertainties in mass and inertia moments without the need for prior knowledge, providing effective control even with varying system parameters. An additional component of the system is a disturbance observer, which is used to manage and mitigate the impact of time-varying external disturbances. A similar solution was presented by Gao et al. in their paper [29], which deals with path tracking by a UAV using a sliding mode based on an angle constraint with convergence in finite time, so that it can fly to a target autonomously.

Analysing the literature from the more general aspect of controller applications, it can be seen that PID controllers [10,11,12,14,15,30,31,32,33] have a dominant role in various engineering applications. Classical PID controllers have the following advantages: they are simple in structure, do not rely solely on mathematical models, and are easy to implement in engineering. However, when the internal system parameters are perturbed and the external environment changes, a pre-adapted controller often shows poor adaptability [13]. The solution to these problems is to adapt the controller parameters using known methods or develop new ones, i.e., using artificial neural networks [14,31,33], fuzzy logic [15,30,34], back-propagation techniques [31,33,35], and others [12,13,30,32,36]. For example, Aurelio G. Melo et al. [34] propose a novel strategy based on a fuzzy gain scheduling mechanism to adjust the PID controller to stabilise both position and height control. Salwa and Krzysztofik in their paper [36] took a new approach to selecting PID controller parameters for UAVs during programmed flights. The authors then proceeded to select gains using optimisation methods. The following methods were used: fmincon, fminimax, lsqnonlin, and fminsearch. The proposed method is an alternative to adaptive control, which requires a significant amount of system identification and parameter tuning. Kownacki C. and Ambroziak L. [30] presented a PID controller combined with the artificial potential field method, which interacts with the integral and differential components. The authors showed that this controller design, which they called non-linear PID, improves control response by suppressing overshoot and minimising steady-state error. Of particular interest are PID controller tuning methods using artificial neural networks. For example, Omar Rodríguez-Abreo et al. [31] presented a self-adjusting PID controller based on a back-propagation artificial neural network. The network calculates the appropriate gains according to the desired output, i.e., the desired dynamic response, which consists of a transient and a stationary step response of the system. In addition to using the error to train the network, the authors also used the maximum desired values of overshoot, settling times, and stationary errors as input for the neural network. On the other hand, Marino and Neri [33] presented the performance of a recurrent neural network for tuning a PID controller and empirically investigated recurrent multilayer perceptrons to synthesise a PID controller and its gains by specifying proportional, integral, and derivative actions.

The novelty of the present study lies in the development of a pitch channel control system for a UAV moving at speeds close to and higher than the speed of sound, which has not been considered in the literature thus far. In addition, a proprietary approach was introduced in the synthesis of the control system by optimising the controller parameters using a recurrent neural network. The basis for the research presented in this paper was the study of [31,33] that presents the use of a neural network PID to control simple objects such as a DC motor or an inertial member. A UAV is much more complex object to control than a DC motor. Therefore, in our opinion, as supported by the literature review, it is valuable to develop a PID controller with a neural network, learning through back propagation, in the aspect of UAV pitch channel control.

## 4. Control System Design

The PID controller was selected to guide the UAV along a defined trajectory due to its versatility, widespread use, and proven effectiveness in autopilot systems, as evidenced by numerous studies: [10,11,12,14,15,30,31,32,33]. Its mathematical simplicity allows for straightforward software implementation, and its parameters can be tuned to match the system’s characteristics, offering significant adaptability. Moreover, the PID controller operates in real time, providing rapid and effective stabilization of dynamic systems while minimizing control errors. Additionally, well-established methods for determining the optimal PID settings are thoroughly documented in the literature.

The PID controller’s operation relies on processing the error signal e(t) through three fundamental components: the proportional term *P*, which applies a gain to the error signal; the integral term *I*, which accumulates the error over time to address steady-state deviations; and the derivative term *D*, which predicts future error behaviour by analysing the rate of change. This combination enables the PID controller to deliver precise and reliable control performance.

The operator transmittance of an ideal PID controller has the following form:

(17) $$G_{r}\left(s\right)=k_{p}\left(1+\frac{1}{sT_{I}}+sT_{D}\right)$$

where: $k_{p}$—proportional gain coefficient, $T_{I}$—integration time constant, $T_{D}$—differentiation time constant.

In practice, it is impossible to achieve perfect differentiation over the entire frequency range. As a result, practical controllers incorporate an inertial component that limits the impact of differentiation at higher frequencies. This inertial element acts as a low-pass filter, governed by a carefully chosen filter parameter *N*. Accordingly, a real PID controller was implemented for the control system, with a transfer function given by:

(18) $$G_{r}\left(s\right)=k_{p}\left(1+\frac{1}{sT_{I}}+\frac{T_{D}s}{1+\frac{T_{D}}{N}s}\right)$$

where *N* is the filtration coefficient.

The next step in synthesizing the control system for the task outlined in this article was to determine the controller settings (kp, $T_{I}$ and $T_{D}$), addressing the research problem discussed in Section 2.2. Initially, the controller parameters were calculated using the Ziegler–Nichols II method, as detailed in Section 4.1. Subsequently, the settings were optimized through the author’s novel approach, which involves determining the controller parameters using a recursive neural network, as described in Section 4.2.

### 4.1. Path Tracking Control System with Classic PID Controller

**Figure 3:**
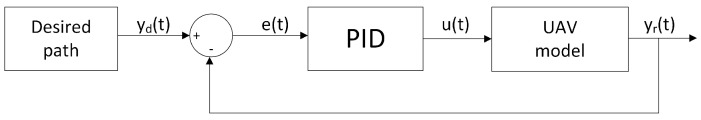
Control system block diagram ($y_{d}\left(t\right)$—desired flight height (input), $y_{r}\left(t\right)$—simulation determined height (output), e(t)—error determined from equation e(t) = $y_{d}\left(t\right)$−$y_{r}\left(t\right)$, u(t)—control signal (angle $\delta_{w}$).

In this case study, a control system with a controller in real form (Equation (15)) was used, which is shown in the Figure 3:

The control strategy focuses on precisely regulating the UAV’s flight altitude. The control signal u(t), represented as the steering angle $\delta_{w}$, is generated by the PID controller based on the error signal e(t), which is the difference between the desired flight height $y_{d}\left(t\right)$ and the actual flight altitude $y_{r}\left(t\right)$. A change in the steering angle $\delta_{w}$ induces a pitching moment $M_{Z_{1}}$, as described by Equations (12) and (13). This pitching moment affects the UAV airframe, leading to a change in the pitch angle ϑ according to Equations (3) and (4). Consequently, the pitch angle influences the angle of attack, as defined by Equation (5). The angle of attack generates a lift force, described by Equations (10) and (11), which alters the inclination angle of the velocity vector, as given by Equation (2). Ultimately, these dynamics result in a change in flight height, governed by Equation (7), which the control system continuously adjusts to maintain the desired trajectory.

The control signal u(t) coming out of the used PID controller has the following form:

(19) $$u\left(t\right)=k_{p}\left(e\left(t\right)+\frac{1}{T_{I}}\int_{0}^{t}e\left(t\right)dt+Ne{\left(t\right)}^{-\frac{tN}{T_{D}}}\right)$$

and is then constrained to $\delta_{max}$ = ±15°, as specified by the limitations of the rudders in real supersonic UAVs.

**Figure 4:**
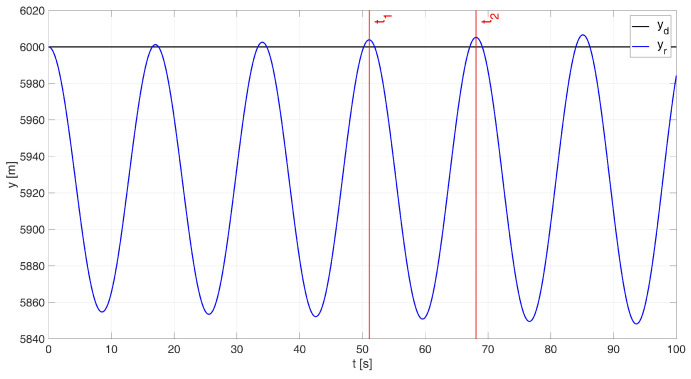
The stability limit of the system under investigation.

The next stage in synthesizing the control system involved selecting the controller gains. Initially, the engineering approach based on the Ziegler–Nichols II method [32,33] was used to determine the controller parameters. The Ziegler–Nichols II method involves using a purely proportional controller, after which the gain of this controller is gradually increased until the system reaches the stability limit. Achieving the stability limit for a system with such high dynamics proved to be both challenging and time-consuming. After several attempts, the control system was successfully tuned to the stability limit, as shown in Figure 4. The critical gain value $k_{kr}$ = −0.0015 s and the oscillation period $T_{osc}$ = 18.24 s were then recorded.

Finally, the following formulae were used:

(20) $$\begin{array}{ccc} k_{p}=0.6k_{kr}, & T_{I}=0.5T_{osc}, & T_{D}=0.125T_{osc} \end{array}$$

The gain values of the PID controller were determined as follows:

(21) $$\begin{array}{ccc} k_{p}=-0.0009, & T_{I}=0.1097, & T_{D}=2.28 \end{array}$$

### 4.2. Path Tracking Control System with Neural Network PID Controller

Controlling UAV flight at high speeds, particularly near or above the speed of sound, presents unique challenges for the control system. These conditions introduce complex non-linear dynamics and time-varying uncertainties, which make traditional control methods, such as basic PID, less effective. To address this, the control strategy outlined earlier was enhanced by integrating a PID controller with a recurrent neural network (RNN) to optimize performance under these demanding conditions. The inclusion of the RNN enables the controller to adapt and learn over time, thereby improving its resilience to disturbances and dynamic changes in the system.

**Figure 5:**
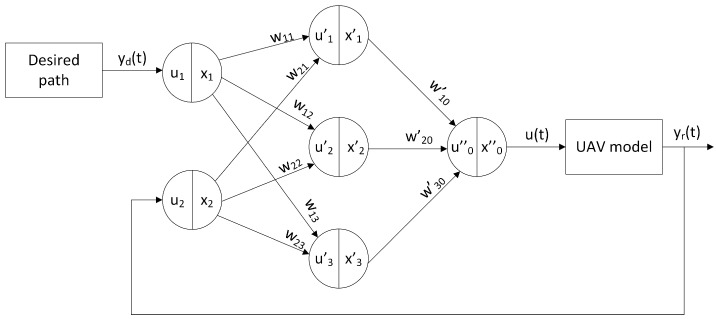
Structure of the control system with a neural network PID controller.

A neural network (NN) is a non-linear mathematical structure composed of a collection of interconnected neurons or nodes. The properties of a neural network are defined by its topology and the characteristics of its nodes. In this section, we describe the modelling of a PID controller tuned using an RNN, as illustrated in Figure 5. The RNN was selected for optimizing the controller parameters due to its ability to handle the dynamic and time-dependent nature of UAV flight. Unlike traditional optimization methods, an RNN can learn from historical data and adjust its predictions accordingly. This capability is particularly valuable in UAV control, where system behaviour evolves over time due to changes in altitude, speed, and external disturbances. RNNs can capture temporal dependencies and adapt to changes in the system state, resulting in improved control accuracy and reduced response times. Moreover, RNNs are well-suited to problems requiring sequential decision-making, which is common in trajectory tracking and stabilization tasks. They are capable of processing long sequences (e.g., an extended flight trajectory) and can operate on data of varying lengths (e.g., different flight durations). A PID controller integrated with a neural network will be referred to as a PIDNN.

The used network (Figure 5) consists of three layers. The input layer contains two neurons: $x_{1}$, which receives the reference signal $y_{d}\left(t\right)$, and $x_{2}$, which receives the system output signal $y_{r}\left(t\right)$. The middle layer consists of three neurons, a proportional neuron ($x_{1}^{\prime }$), an integral neuron ($x_{2}^{\prime }$), and a derivative neuron ($x_{3}^{\prime }$), each of which performs one of the three fundamental control operations: amplification, integration, and differentiation. The third layer combines these three control components into a single output $x_{o}^{\prime \prime }$.

The weights of the individual neural connections were defined as follows:

(22) $$\begin{array}{ccccc} w_{1j}=1, & w_{2j}=-1, & w_{10}=k_{p}, & w_{20}=\frac{1}{T_{I}}, & w_{30}=T_{D} \end{array}$$

By analyzing the signal flow structure of the neural network shown in Figure 5, from the input layer to the output layer, the functions describing the neurons in the middle layer can be expressed as follows [33]:

(23) $$x_{1}^{\prime }\left(t\right)=u_{1}^{\prime }\left(t\right)=w_{11}x_{1}\left(t\right)+w_{21}x_{2}\left(t\right)=y_{d}\left(t\right)-y_{r}\left(t\right)=e\left(t\right)$$

(24) $$x_{2}^{\prime }\left(t\right)=\int_{0}^{t}u_{2}^{\prime }\left(t\right)dt=\int_{0}^{t}\left[w_{12}x_{1}\left(t\right)+w_{22}x_{2}\left(t\right)\right]dt=\int_{0}^{t}e\left(t\right)dt$$

(25) $$x_{3}^{\prime }\left(t\right)=\frac{du_{3}^{\prime }\left(t\right)}{dt}=\frac{d[w_{12}x_{1}\left(t\right)+w_{22}x_{2}\left(t\right)]}{dt}=\frac{de(t)}{dt}$$

as well as a function describing the output neurone as

(26) $$\begin{array}{c} x_{0}^{\prime \prime }\left(t\right)=u_{0}^{\prime \prime }\left(t\right)=\sum_{j=1}^{3}w_{j0}^{\prime }x_{j}^{\prime }\left(t\right)=w_{10}^{\prime }x_{1}^{\prime }\left(t\right)+w_{20}^{\prime }x^{\prime }2\left(t\right)+w_{30}^{\prime }x_{3}^{\prime }\left(t\right)\\ =k_{p}e\left(t\right)+\frac{1}{T_{I}}\int_{0}^{t}e\left(t\right)dt+T_{D}\frac{de(t)}{dt} \end{array}$$

The neural network used is an automatic tuning method based on functional analysis in successive simulation trials. The tuning consists of changing the weights of the neurone connections using the backpropagation algorithm (Algorithm 1) [33]. This algorithm changes the weights $w_{10}$, $w_{20}$, $w_{30}$, initialised with fixed values, which were previously determined by the Z-N method II, in successive simulation runs.
**Algorithm 1** Backpropagation**Input:** $y_{d}\left(m\right)$, $y_{d}\left(m+1\right)$, $y_{r}\left(m\right)$, $y_{r}\left(m+1\right)$, u(m−1), u(m), $x_{1}\left(m\right)$, $x_{1}\left(m+1\right)$, $x_{2}\left(m\right)$, $x_{2}\left(m+1\right)$, $x_{3}\left(m\right)$, $x_{3}\left(m+1\right)$  1:Δ←$\frac{2(y_{d}\left(m\right)\;-\;y_{r}\left(m\;+\;1\right)\;*\;(y_{r}\left(m\right)\;-\;y_{r}\left(m\;+\;1\right)}{u(m\;-\;1)\;-\;u(m)}$  2:Δ1←$\Delta k_{p}\frac{x_{1}\left(m\;+\;1\right)\;-\;x_{1}\left(m\right)}{\left(y_{d}\left(m\right)\;-\;y_{r}\left(m\right)\right)\;-\;\left(y_{d}\left(m\right)\;-\;y_{r}\left(m\;+\;1\right)\right)}$  3:Δ2←$\Delta T_{I}\frac{x_{2}\left(m\;+\;1\right)\;-\;x_{2}\left(m\right)}{\left(y_{d}\left(m\right)\;-\;y_{r}\left(m\right)\right)\;-\;\left(y_{d}\left(m\right)\;-\;y_{r}\left(m\;+\;1\right)\right)}$  4:Δ3←$\Delta T_{D}\frac{x_{3}\left(m\;+\;1\right)\;-\;x_{3}\left(m\right)}{\left(y_{d}\left(m\right)\;-\;y_{r}\left(m\right)\right)\;-\;\left(y_{d}\left(m\right)\;-\;y_{r}\left(m\;+\;1\right)\right)}$  5:**for** i←0 to *m* **do**  6:      J(1)←$J\left(1\right)-\left(\Delta \frac{x_{1}\left(m\right)}{m}\right)$  7:      J(2)←$J\left(2\right)-\left(\Delta \frac{x_{2}\left(m\right)}{m}\right)$  8:      J(3)←$J\left(3\right)-\left(\Delta \frac{x_{3}\left(m\right)}{m}\right)$  9:      J(4)←$J\left(4\right)-\left(\Delta 1\frac{y_{d}\left(m\right)}{m}\right)-\left(\Delta 2\frac{y_{d}\left(m\right)}{m}\right)-\left(\Delta 3\frac{y_{d}\left(m\right)}{m}\right)$10:      J(5)←$J\left(5\right)-\left(\Delta 1\frac{y_{r}\left(m\right)}{m}\right)-\left(\Delta 2\frac{y_{r}\left(m\right)}{m}\right)-\left(\Delta 3\frac{y_{r}\left(m\right)}{m}\right)$11:**end for**12:$k_{p}$←$k_{p}-J\left(1\right)n_{o}$13:$T_{I}$←$T_{I}-J\left(2\right)n_{o}$14:$T_{D}$←$T_{D}-J\left(3\right)n_{o}$15:$k_{1}$←$k_{1}-J\left(4\right)n_{i}$16:$k_{2}$←$k_{2}-J\left(2\right)n_{i}$17:*m*←m+118:**return** $k_{p}$, $T_{I}$, $T_{D}$, $k_{1}$, $k_{2}$

The goal of the backpropagation algorithm is to create a database that links PID gains to their corresponding dynamic responses. In this way, the algorithm searches for gains that produce a specific desired dynamic behaviour. For each sample *m*, the proposed algorithm monitors the system output signal $y_{r}\left(t\right)$, the controller output signal u(t), and the individual neuron signals $x_{1}^{\prime },x_{2}^{\prime }$ and $x_{3}^{\prime }$. It then compares these outputs to the input reference signal $y_{d}\left(t\right)$ and calculates the difference between the actual outputs and the desired output. The network error is defined as the discrepancy between the actual and desired values. The individual weights are updated by backpropagating the calculated error. This process is repeated iteratively to minimize the error. Ultimately, the algorithm learns by sampling these signals.

A crucial factor in the proper functioning of the backpropagation algorithm is the learning rate, a parameter that determines the step size by which the weights are adjusted in the direction opposite to the error gradient. The learning rate of the algorithm is controlled by two coefficients: $n_{i}$, the learning rate for the input neurons, and $n_{o}$, the learning rate for the output neurons [33]. If the learning rate is too small, the learning process will be very slow, as the weights change only slightly with each iteration. Conversely, if the learning rate is too large, it can cause instability in the system.

In the research described here, the learning rate was determined empirically using established methods for selecting this parameter. Initially, a grid search approach was employed, where several values within a predefined range were tested. The tested values included {1, 0.1, 0.001, 0.0001, 0.00001, 0.000001}. The range that produced the best simulation results was then selected. Following this, dynamic scaling was applied, where the learning rate was adjusted by a specific amount after a fixed number of iterations.

## 5. Simulation Results and Discussion

Path tracking simulations were conducted in the MATLAB/Simulink environment and included two flight start heights and flights along a desired path at subsonic speeds, at close to the speed of sound, and at supersonic speeds.

The following trajectory and movement variants have been proposed:

- Start flight at height $y_{0}$ = 6000 m; flight at steady speed (0.65 Ma, 0.9 Ma and 2 Ma); descent to height $y_{c}$ = 4 m, flight to the end point at height 4 m, maintaining a steady speed;
- Start flight at height $y_{0}$ = 200 m; flight at steady speed (0.65 Ma, 0.9 Ma and 2 Ma); descent to height $y_{c}$ = 4 m, flight to the end point at height 4 m maintaining a steady speed;

Simulations were conducted using the classic PID controller and then the PIDNN controller for path tracking in both cases mentioned above.

The quality of control during the path tracking by the UAV was evaluated for the following:

- Values of the functional for the Integral Time Absolute Error (ITAE) criterion:
  (27) $$ITAE=\int_{0}^{t}\left(t|e\left(t\right)|\right)dt$$
- Regulation time $t_{r}$ (the time after which the value of the error e(t) will not be greater than 4 m);
- Steady state error value $e(t_{final})$.

The results are presented in the form of a comparison between a reference trajectory, a simulation trajectory plot, and a height error plot.

### 5.1. Simulation of Path Tracking Using a Classic PID Controller

The parameters $k_{p}$, $T_{I}$, and $T_{D}$ of the classical PID controller were determined from the Equation (20) and took the following values:

(28) $$\begin{array}{ccc} k_{p}=-0.0009, & T_{I}=0.1097, & T_{D}=2.28 \end{array}$$

**Figure 6:**
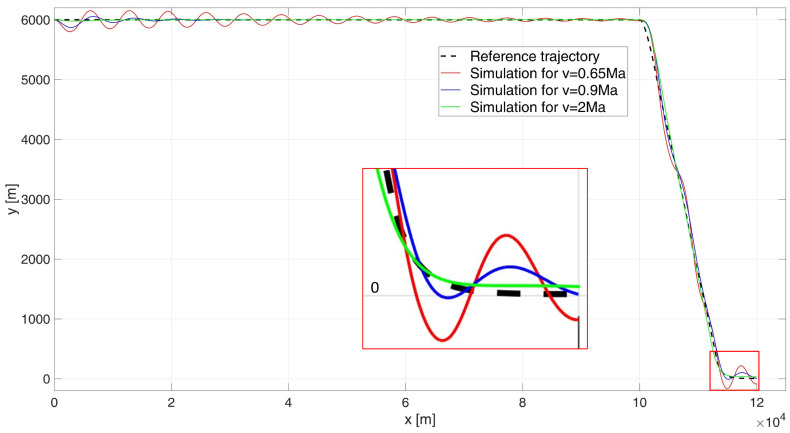
Flight path of a UAV controlled by a classical PID–start at 6000 m height.

**Figure 7:**
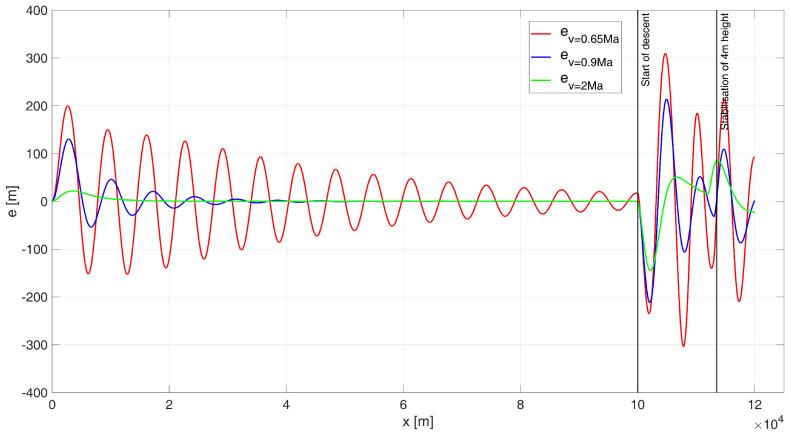
Height errors during flight of a UAV controlled by a classic PID, starting at an altitude of 6000 m.

The result of the simulation is the flight path shown in Figure 6 and the height errors shown in Figure 7.

Analysing the UAV’s flight path shown in Figure 6, it is evident that none of the simulation variants meet the control requirements. For flight at a subsonic speed of 0.65 Ma, large and prolonged oscillations occur, making it impossible to accurately determine the regulation time during the initial flight phase. Additionally, it is observed that the flight altitude drops significantly below the surface of the water (or land), leading to the destruction of the UAV.

For flight near the speed of sound, at 0.9 Ma, a notable improvement in control quality is observed. The simulated flight path closely follows the desired trajectory, with a control time of $t_{r_{0.9}}$ = 104.8 s. For a supersonic flight speed of 2 Ma, the path overlaps significantly with the desired trajectory, and the regulation time is $t_{r_{2}}$ = 16.6 s. During the simulation, the ITAE indicator values were determined as follows: $ITAE_{0.65}$ = $8.87\times 10^{6}$, $ITAE_{0.9}$ = $1.98\times 10^{9}$, $ITAE_{2}$ = $2.33\times 10^{5}$.

When analysing the error plot (Figure 7), it is evident that for both 0.9 Ma and 2 Ma flight speeds, the high-altitude flight proceeds correctly. However, oscillations are observed during both the descent phase to a height of 4 m and the stabilization phase at this altitude for each case. In the simulations, the absolute values of the final errors were determined as follows: for the 0.65 Ma flight speed, $e_{0.65}\left(t_{final}\right)$ = 92.41 m; for the 0.9 Ma flight speed, $e_{0.9}\left(t_{final}\right)$ = 0.28 m (although the earlier drop below water or ground level disqualifies this case); and for the 2 Ma flight speed, $e_{2}\left(t_{final}\right)$ = 27.58 m.

**Figure 8:**
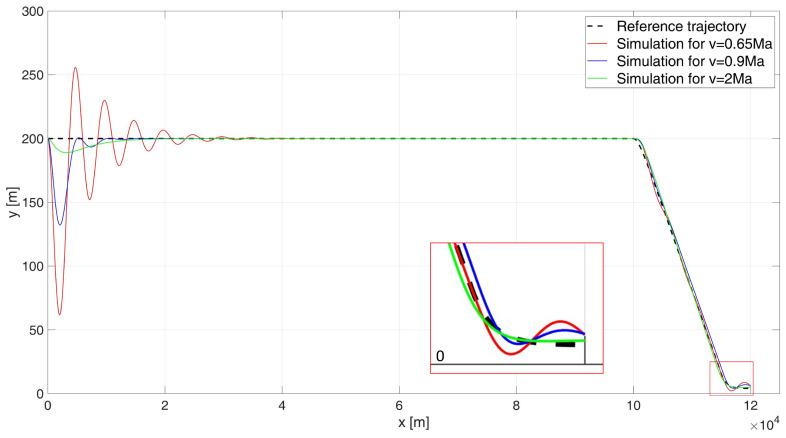
Flight path of a UAV controlled by a classical PID–start at 200 m height.

When analysing the UAV’s flight path shown in Figure 8, it is evident that the control system performs better than in the 6000 m drop case, as the UAV did not hit the water surface in any of the simulation variants. For subsonic flight at 0.65 Ma, large but diminishing oscillations are observed. The regulation time is $t_{r_{0.65}}$ = 102.4 s. For flights near the speed of sound, at 0.9 Ma, a significant improvement in control quality is noted. The simulated flight path closely matches the desired trajectory, with a control time of $t_{r_{0.9}}$ = 27.8 s. For a supersonic flight speed of 2 Ma, the simulation-determined trajectory closely overlaps with the desired path. The regulation time for this case is $t_{r_{2}}$= 13.4 s. During the simulation, the ITAE indicator values were determined as follows: $ITAE_{0.65}$ = $1.67\times 10^{5}$, $ITAE_{0.9}$ = $3.12\times 10^{4}$, $ITAE_{2}$ = $5.27\times 10^{3}$.

**Figure 9:**
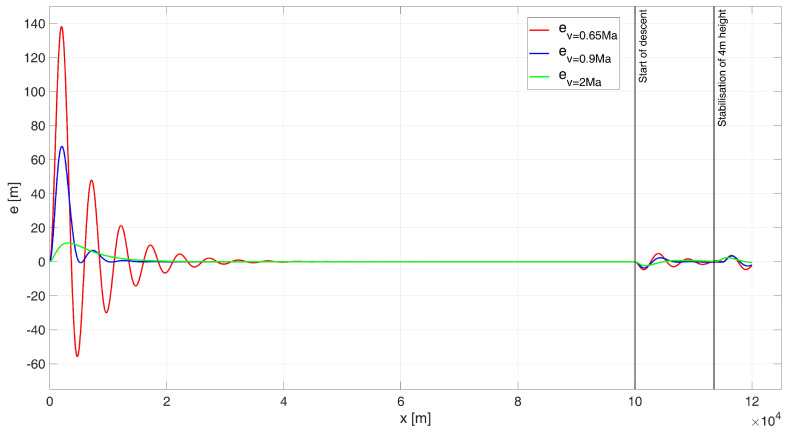
Height errors during flight of a UAV controlled by a classic PID, starting at an altitude of 200 m.

When analysing the error plot (Figure 9), it is evident that in all simulation variants, the high-altitude flight proceeds correctly. During both the descent phase to a height of 4 m and the stabilization phase at this altitude, small and diminishing oscillations were observed in each case. In the simulations, the absolute values of the steady-state errors were determined as follows: for a speed of 0.65 Ma, $e_{0.65}\left(t_{final}\right)$ = 2.06 m; for a speed of 0.9 Ma, $e_{0.9}\left(t_{final}\right)$ = 2.24 m; and for a speed of 2 Ma, $e_{2}\left(t_{final}\right)$ = 0.87 m.

### 5.2. Simulation of Path Tracking Using a Neural Network PID Controller

**Figure 10:**
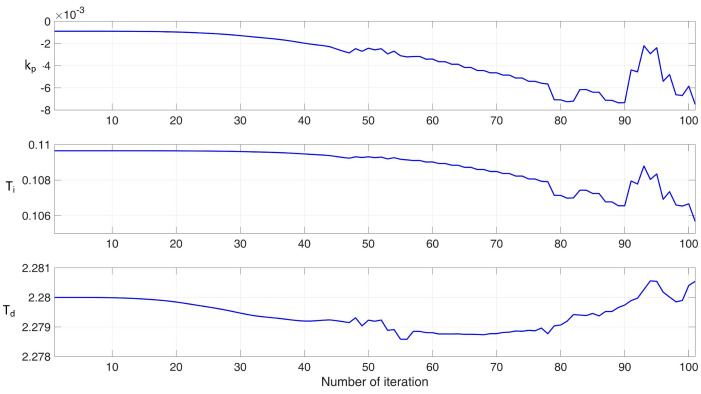
The changes in parameters $k_{p}$, $T_{I}$, $T_{D}$ during the learning of the neural network.

During the tests, the number of algorithm iterations was set to 100, the signal sampling rate was set to 0.001, the learning rate for the output neurons $n_{o}$ = 0.00035, and the learning rate for the input neurons $n_{i}$ = 1. These values for $n_{o}$ and $n_{i}$ were determined experimentally, and mathematical methods for their selection will be explored in future work. The initial gains, as determined using the Ziegler–Nichols II method, were used as starting values. The variations in the parameters $k_{p}$, $T_{I}$ and $T_{D}$ during the neural network’s learning process are shown in Figure 10.

The parameters of the PIDNN controller determined with the smallest value of the ITAE indicator (Equation (24)) took the following values:

(29) $$\begin{array}{ccc} k_{p}=-0.0152, & T_{I}=0.1052, & T_{D}=2.2696 \end{array}$$

**Figure 11:**
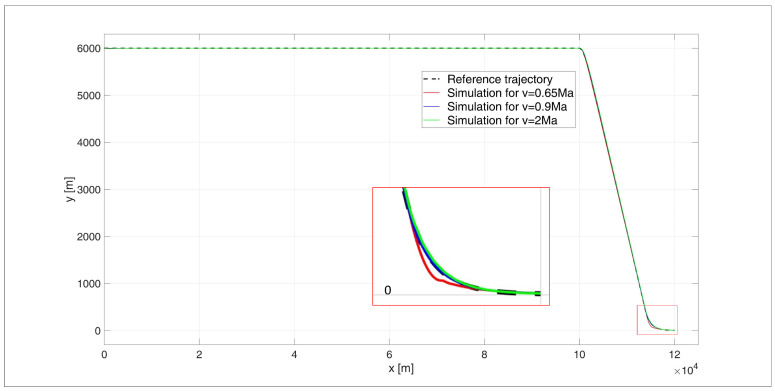
Flight path of a UAV controlled by a PIDNN, starting at an altitude of 6000 m.

**Figure 12:**
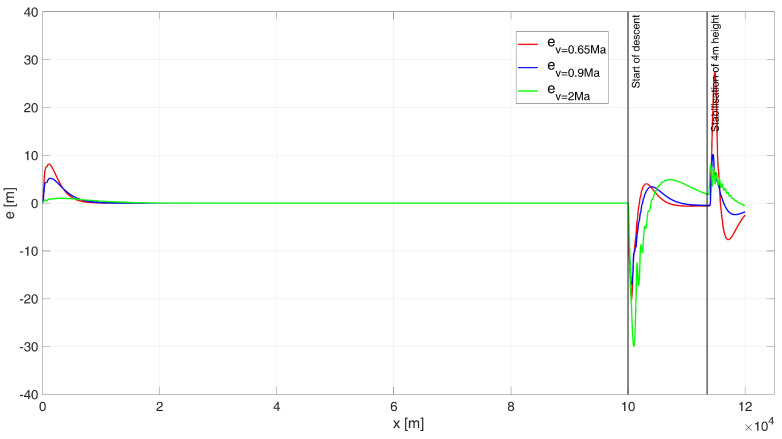
Height errors during flight of a UAV controlled by a PIDNN, starting at an altitude of 6000 m.

**Figure 13:**
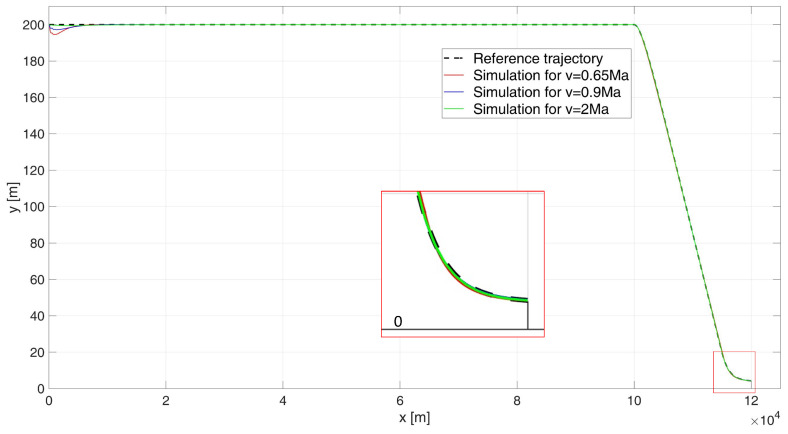
Flight path of a UAV controlled by a PIDNN- start at 200 m height.

**Figure 14:**
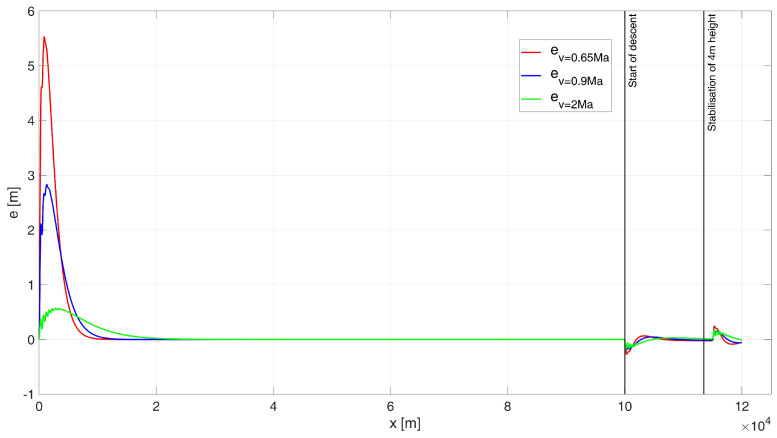
Height errors during flight of a UAV controlled by a PIDNN, starting at an altitude of 200 m.

The simulation results were as follows: for a starting altitude of 6000 m, the flight path is shown in Figure 11 and the height errors are shown in Figure 12; for a start height of 200 m, the flight path is shown in Figure 13 and the height errors are shown in Figure 14.

When analysing the UAV’s flight path, as shown in Figure 11, it is evident that there is a very good overlap between the desired trajectory and the simulated trajectory for all tested flight speed variants, as reflected by the ITAE indicator values: $ITAE_{0.65}$ = $1.62\times 10^{5}$, $ITAE_{0.9}$ = $6.04\times 10^{4}$, $ITAE_{2}$ = $2.36\times 10_{4}$. Only during the subsonic flight at 0.65 Ma was a noticeable path tracking error observed at the beginning of the flight stabilization phase at an altitude of 4 m. However, the error was small enough that the UAV did not hit the water surface. The regulation time for this case was $t_{r_{0.65}}$ = 13.26 s. For flight near the speed of sound at 0.9 Ma, the regulation time was $t_{r_{0.9}}$ = 9 s. For supersonic flight at 2 Ma, the regulation time was determined as $t_{r_{2}}$= 0 s, since the UAV never deviated by more than 4 m in height.

When analysing the error plot (Figure 12), it can be observed that for the 2 Ma flight, a large transient error occurred at the beginning of the descent manoeuvre, though this is not that relevant to the task at hand. For the 0.65 Ma flight, a significant transient error occurred at the start of the flight stabilization manoeuvre at 4 m. However, the nature of this overshoot did not cause the UAV to hit the water surface. In the simulations, the absolute values of the steady-state errors were determined as follows: for a speed of $e_{0.65}\left(t_{final}\right)$ = 2.09 m; for a speed of 0.9 Ma, $e_{0.9}\left(t_{final}\right)$ = 1.9 m; and for a speed of 2 Ma, $e_{2}\left(t_{final}\right)$ = 1.44 m.

When analyzing the UAV’s flight path shown in Figure 13, it is clear that there is an excellent overlap between the desired path and the path determined by the simulation for all flight speed variants, as evidenced by the ITAE indicator values: $ITAE_{0.65}$ = $2.77\times 10^{3}$, $ITAE_{0.9}$ = $1.33\times 10^{3}$, $ITAE_{2}$ = 277.63. For the subsonic flight, the regulation time is $t_{r_{0.65}}$ = 9.5 s. For flight near the speed of sound (0.9 Ma), the regulation time is $t_{r_{0.9}}$ = 0 s, and for supersonic flight at 2 Ma, the regulation time was also determined to be $t_{r_{2}}$ = 0 s, since the UAV never experienced a height error greater than 4 m.

When analysing the error plot (Figure 14), it can be seen that for all the cases studied, there is a transient error at the beginning of the flight (which is not very significant from the point of view of the task at hand), and a slight transient error at the beginning of the descent manoeuvre and at the beginning of the flight stabilisation manoeuvre at 4 m (which does not result in hitting the water surface). In the simulations, the absolute values of the terminal errors were determined: for speed 0.65 Ma equal to $e_{0.65}\left(t_{final}\right)$ = 0.24 m, for speed 0.9 Ma equal to $e_{0.9}\left(t_{final}\right)$ = 0.33 m, and for speed 2 Ma equal to $e_{2}\left(t_{final}\right)$ = 0.01 m.

When analysing the error plot (Figure 14), it can be seen that for all the cases studied, there is a transient error at the beginning of the flight (which is not highly significant from the perspective of the task at hand), as well as a slight transient error at the start of the descent manoeuvre and during the flight stabilization manoeuvre at 4 m. However, these errors do not result in the UAV hitting the water surface. In the simulations, the absolute values of the terminal errors were determined as follows: for a speed of 0.65 Ma $e_{0.65}\left(t_{final}\right)$ = 0.24 m; for a speed of 0.9 Ma, $e_{0.9}\left(t_{final}\right)$ = 0.33 m; and for a speed of 2 Ma, $e_{2}\left(t_{final}\right)$ = 0.01 m.

### 5.3. Discussion

A comparison of the evaluation parameters for the control system investigated in this paper is presented in Table 1, Table 2, Table 3, Table 4, Table 5 and Table 6.

Analyzing the results summarized in the Table 1, Table 2 and Table 3, it is evident that the control system for guiding the UAV along the desired path, with PID controller parameters determined using the Ziegler–Nichols II method, does not meet the requirements. The best result was obtained for the flight at 0.9 Ma, for which the control system parameters are compared in Table 2. However, despite a small transient error, the UAV crashed into the water before reaching the end point of the trajectory, which also occurred in the other two cases. This was caused by the significant increase in descent speed due to the long descent distance. As a result, the controller was unable to manage the object’s dynamics within a short period. Furthermore, each tested case is characterized by a long regulation time, which is highly unfavourable when the trajectory’s endpoint is a short distance from the starting point (e.g., tens or hundreds of kilometers), as the UAV may not have enough time to stabilize its flight.

In the case of a UAV starting its flight at an altitude of 200 m and following the desired path with control by a classical PID controller, the transient error in each case is significantly smaller than for a starting altitude of 6000 m, as summarized in the Table 4, Table 5 and Table 6. However, the control system using the classic PID controller is still characterized by very long control times.

A comparison of controller parameters ($k_{p}$, $T_{i}$ and $T_{d}$) obtained using the Ziegler–Nichols II method (PID) and those optimized with a neural network (PIDNN) is shown in the Table 7. The results demonstrate a substantial adjustment to the proportional gain, accompanied by minor refinements to the integral and derivative gains. After optimizing the controller gains using the neural network, a significant improvement in the control system’s performance is observed. In every analysed case, the control system with the PIDNN controller is characterised by considerably shorter regulation times compared to the PID controller, as well as significantly smaller transient and steady-state errors. Most importantly, in each case, the UAV followed the desired path accurately, as confirmed by the ITAE indicators.

Furthermore, during the neural network’s learning process, it was observed that the parameter $n_{i}$ does not significantly affect the learning time or quality for the case studied. This is because the input neurons simply pass the signal to the next layer and do not adjust their own weights, as their primary function is to represent the input data. For the input layer, proper scaling and standardization of the input data, as well as appropriate initialization of the weights, are crucial.

## 6. Conclusions

This paper presents a control system for UAV flight along a desired path, focusing specifically on the pitch control channel in the control plane. A mathematical model of the UAV, formulated in the Earth’s coordinate system, is provided. Both the model and the control system were implemented in the MATLAB/Simulink environment. The control system is based on a PID controller, with its gains initially determined using the Ziegler–Nichols II method. This paper proposes the optimisation of these parameters using a backpropagation neural network. Simulation studies were conducted for two trajectory variants: one with the UAV starting at 6000 m, descending to 4 m, and flying at a steady speed, and the other with the UAV starting at 200 m, descending to 4 m, and flying at a steady speed. For each variant, the UAV’s flight was analysed at subsonic speeds, close to the speed of sound, and supersonic speeds.

The results of the simulations demonstrate that optimizing the PID controller gains with a neural network significantly reduced the regulation time (in some cases, by up to 100%) and greatly minimized the final errors. The reduction in the ITAE indicator by at least one order of magnitude in each case confirms the improved accuracy of path tracking achieved using the PIDNN controller, validating the effectiveness of this approach.

## Figures and Tables

**Table 1:** Evaluation parameters of the control system for the case of a flight starting at an altitude of 6000 m and a speed of 0.65 Ma.

		$t_{r}$ [s]	$e_{\mathrm{final}}$ [m]	ITAE
V = 0.65 Ma	PID	not specified	92.41	$8.87\times 10^{6}$
	PIDNN	13.26	2.03	$1.62\times 10^{5}$

**Table 2:** Evaluation parameters of the control system for the case of a flight start at an altitude of 6000 m and a speed of 0.9 Ma.

		$t_{r}$ [s]	$e_{\mathrm{final}}$ [m]	ITAE
V = 0.9 Ma	PID	104.8	0.28	$1.98\times 10^{6}$
	PIDNN	9	1.9	$6.06\times 10^{4}$

**Table 3:** Evaluation parameters of the control system for the case of a flight starting at an altitude of 6000 m and a speed of 2 Ma.

		$t_{r}$ [s]	$e_{\mathrm{final}}$ [m]	ITAE
V = 2 Ma	PID	16.6	27.58	$2.33\times 10^{5}$
	PIDNN	0	1.44	$2.36\times 10^{4}$

**Table 4:** Evaluation parameters of the control system for the case of a flight starting at an altitude of 200 m and a speed of 0.65 Ma.

		$t_{r}$ [s]	$e_{\mathrm{final}}$ [m]	ITAE
V = 0.65 Ma	PID	102.4	2.06	$1.67\times 10^{5}$
	PIDNN	9.5	0.24	$2.77\times 10^{3}$

**Table 5:** Evaluation parameters of the control system for the case of a flight starting at an altitude of 200 m and a speed of 0.9 Ma.

		$t_{r}$ [s]	$e_{\mathrm{final}}$ [m]	ITAE
V = 0.9 Ma	PID	27.8	2.24	$3.112\times 10^{4}$
	PIDNN	0	0.33	$1.36\times 10^{3}$

**Table 6:** Evaluation parameters of the control system for the case of a flight starting at an altitude of 200 m and a speed of 2 Ma.

		$t_{r}$ [s]	$e_{\mathrm{final}}$ [m]	ITAE
V = 2 Ma	PID	13.4	0.87	$2.36\times 10^{4}$
	PIDNN	0	0.01	277.63

**Table 7:** Comparison of controller tunings determined by the Ziegler–Nichols II method and optimized using a neural network.

	$k_{p}$	$T_{i}$	$T_{d}$
PID	−0.0009	0.1097	2.2800
PIDNN	−0.0152	0.1052	2.2696

## Data Availability

Data are contained within the article.

## Abbreviations

The following abbreviations are used in this manuscript:

*m*	mass
$I_{Z_{1}}$	moment of inertia
*V*	linear velocity
$\omega_{Z_{1}}$	angular rate
*P*	the force of the thrust
$X_{a}$	drag force
$Y_{a}$	lifting force
*G*	gravity
$M_{Z_{1}}$	tilting torque
Θ	angle of the inclination of the velocity vector
α	angle of attack
ϑ	UAV pitch angle
$x_{Z}$, $y_{Z}$	coordinates of the UAV in the Earth’s coordinate system
$C_{X_{a}}$	drag coefficient
$C_{X_{0}}$, $C_{X_{a}}^{\alpha^{2}}$, $C_{X_{a}}^{{\delta_{w}}^{2}}$	aerodynamic factors related to drag force
$C_{Y_{a}}$	lift coefficients
$C_{Y_{a}}^{\alpha }$, $C_{{Y_{a}}^{\delta_{w}}}$	aerodynamic factors related to lift force
$\delta_{w}$	steering angle
ρ	atmospheric density
*S*	characteristic surface
*L*	characteristic linear parameter
$m_{Z_{1}}$, $m_{Z_{1}}^{\alpha }$, $m_{Z_{1}}^{\delta_{w}}$, $m_{Z_{1}}^{\omega_{Z_{1}}}$	coefficients related to the number of waves
